# In-Cardiome: integrated knowledgebase for coronary artery disease enabling translational research

**DOI:** 10.1093/database/bax077

**Published:** 2017-10-10

**Authors:** Ankit Sharma, Vrushali Deshpande, Madankumar Ghatge, Rajani Kanth Vangala

**Affiliations:** 1 Manipal University, Madhav Nagar, Manipal, Karnataka 576104, India; 2 Bioinformatics and Biostatistics Unit, Thrombosis Research Institute, Narayana Hrudayalaya, 258/A, Bommasandra Industrial Area, Anekal Taluk, Bangalore, Karnataka 560099, India; 3 Transcriptomics and Histopathology Unit, Thrombosis Research Institute, Narayana Hrudayalaya, 258/A, Bommasandra Industrial Area, Anekal Taluk, Bangalore, Karnataka 560099, India; 4 Proteomics and Coagulation Unit, Thrombosis Research Institute, Narayana Hrudayalaya, 258/A, Bommasandra Industrial Area, Anekal Taluk, Bangalore, Karnataka 560099, India

## Abstract

Coronary artery disease (CAD) is a leading cause of death worldwide. Prevention, diagnosis and clinical interventions are dependent on the conventional risk factors like hypertension, diabetes and obesity. However, these conventional risk factors do not completely identify high risk individuals. One major hurdle in the improvement of diagnosis and treatment for CAD is the lack of integration of knowledge from different areas of research like molecular, clinical and drug development. In order to provide comprehensive information from hitherto dispersed data, we developed an integrative knowledgebase called “In-Cardiome or Integrated Cardiome” for all the stake holders in healthcare such as scientists, clinicians and pharmaceutical companies. It is created by integrating 16 different data sources, 995 curated genes classified into 12 different functional categories associated with disease, 1204 completed clinical trials, 12 therapy or drug classifications with 62 approved drugs and drug target networks. This knowledgebase gives the most needed opportunity to understand the disease process and therapeutic impact along with gene expression data from both animal models and patients. The data is classified into three different search categories functional groups, risk factors and therapy/drug based classes. One more unique aspect of In-Cardiome is integration of clinical data of 10,217 subject data from our ongoing Indian Atherosclerosis Research Study (IARS) (6357 unaffected and 3860 CAD affected). IARS data showing demographics and associations of individual and combinations of risk factors in Indian population along with molecular information will enable better translational and drug development research.

**Database URL:**

www.tri-incardiome.org

## Introduction

According to World Health Organization cardiovascular diseases are the number one cause of mortality in the world of which 7.4 million people die due to coronary artery disease (CAD) and majority from low- or middle-income countries (http://www.who.int/mediacentre/factsheets/fs317/en/). Current treatments for disease are based on the various conventional risk factors like hypertension, diabetes and obesity. Concerted efforts are on to reduce the prevalence of these risk factors. However, many CAD patients do not have any of these identifiable risk factors (1, 2). CAD is a multifactorial disease and several researchers are working on unraveling the underlying molecular mechanisms so as to develop potential preventive methods, diagnostics and therapeutic interventions. However, these attempts have not really resulted in overall improvement in prevention or clinical outcomes especially in countries like India where premature CAD is very common. There are few sources of information regarding molecular data (3–5) of genes associated with CAD. However, they lack connectivity between gene-function-drug/therapy and risk factor interplay. These links between functions, genes or drug targets and risk factors are important not only in understanding the disease progression but also in providing much needed opportunities for improved biomarker and drug discovery translational research (6). Development of new interventions and identification of high-risk groups can happen when not just data is shared, but data connectivity is addressed as well. Therefore, our aim was to create a platform for enabling data cross-talk potentially leading to innovative research for better public healthcare worldwide.

Integrated Cardiome (In-Cardiome) knowledgebase was developed primarily to provide a platform for all the stake holders in the healthcare to access the information regarding genes, functions, clinical trials and drugs or therapies and networking of risk factors along with real-time data of their associations in Indian population. Our database can enable improved understanding of molecular pathogenesis, disease progression, current relevant therapies and modulation of molecular pathways by them, and finally how the drug developments in clinical trials are progressing. In-Cardiome is a unified and easy to access knowledgebase, connecting the molecular and clinical worlds for everyone.

## Materials and methods

**Figure 1.**
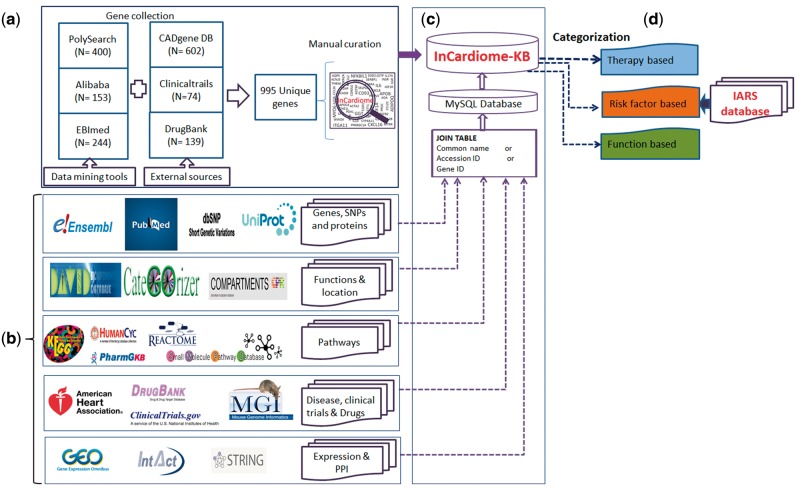
Complete methodology for the construction of In-Cardiome knowledgebase: **(a)** text-mining tools and data sources used for fetching CAD-associated genes, and manual curation. **(b)** Identification of databases for specific information for In-Cardiome gene/proteins. **(c)** Data connectivity and construction of database using MySQL. **(d)** Data classification in In-Cardiome.

The overall methodology is shown in Figure 1 in which following specific steps were followed.

### Data collection and curation

We used three text mining tools namely PolySearch (7), Ali-baba (8) and EBImed (9) for extraction of CAD-associated genes/proteins. 

Terms used for retrieving the CAD-associated gene/protein information were: ATHEROSCLEROTIC CORONARY VASCULAR DISEASE; Arteriosclerosis, Coronary; Arteriosclerotic heart disease; Atherosclerosis, Coronary; Atherosclerotic heart disease; CAD; CORONARY ARTERIOSCLEROSIS; CORONARY SCLEROSIS; Cad; Coronary Artery Diseases; Coronary Atherosclerosis; Coronary arteriosclerosis; Coronary artery arteriosclerosis; CAD; DISEASE CORONARY ARTERY; DISORDER CORONARY ARTERY; Disease of the coronary arteries; Disease, Coronary Artery; Disorder of coronary artery; HEART: CORONARY ARTERY; Ischaemic heart disease; Ischemic heart disease

All the retrieved genes/proteins were manually curated to check their association with CAD.

In the manual curation process, irrelevant gene/protein terms, such as statins, paraoxonase, and carotid intimal medial thickness were removed from the result files. All the filtered genes/proteins were matched with UniProt proteins. Only matched genes/proteins with minimum number of 10 publications proving genes association with CAD were selected. Finally, a unique list of genes/proteins was created after removing redundant entries.

The same term was also used in manually extracting the genes/proteins from ClinicalTrials.gov (10) and DrugBank (11) along with addition of all the genes from CAD Gene Database (3).

CADgene database provides distribution of its genes/proteins in 12 different functional categories. We manually collected gene/protein information from these functional categories and added in In-Cardiome. In the second data source ClinicalTrials.gov, we provided the above mentioned search terms in the advance search option and extracted only completed study with outcome information. Further, we manually searched gene/protein names from the outcomes column of the result file and included them in In-Cardiome gene/protein list. In the third data source called Drug Bank database, we used all the 12 therapy classes as listed by American Heart Association (AHA-http://www.heart.org/HEARTORG/Conditions/HeartAttack/TreatmentofaHeartAttack/Cardiac-Medications_UCM_303937_Article.jsp#.WJxhh9J95dg) as a search term individually in Drug Bank and identified associated drug targets. These targets were further included in In-Cardiome database. We have also used the CAD and its associated terms for manual extraction of target information.

After accumulation of gene lists from each of the tool, all were merged and a unique list of genes was generated by removing redundancy. The chromosomal location of the genes, mutations, literature and single nucleotide polymorphisms (SNPs) were obtained from Ensembl (12) PubMed (13) and dbSNP (14).

The gene expressions were extracted from Gene Expression Ominibus from two different datasets that were studied in home institute (GSE42148, GSE55796) (15–17). Proteins specific information were collected from UniProt (18).

In order to identify the connection between drug targets and In-Cardiome proteins, String database (19) was used. Only experimentally verified human protein–protein interactions were considered with confidence cutoff of > 0.4. For overall In-Cardiome interactome, we fetched both functional and physical interactions from STRING database and INTACT (20) databases (with confidence cutoff of > 0.4).The gene ontology (GO)-based functions were identified using CateGOrizer (21) and David (22) followed by cellular location using Compartments (23). The data about pathways from KEGG (24), PharmaGKB (25), HumanCYC (26), Reactome (27) and Small Molecule Pathway Database (28) (Supplementary Table S1) were added subsequently. The mutant gene phenotype  information was obtained from Mouse Genome Information Database (Supplementary Table S2) (29).

We have also linked PubMed database directly to access the most recent publication information regarding the queried gene/protein. PubMed link has been generated dynamically by the combination of disease terms and regular expressions. Following is an example for dynamic link, created for gene of interest search by user.

(‘Queried_gene/protein_name’)+AND + ((‘coronary + artery + disease’)+OR + (‘ASHD’)+OR + (‘ATHEROSCLEROTIC + CORONARY + VASCULAR + DISEASE’)+OR + (‘Arteriosclerosis’)+OR + (‘Coronary’)+OR + (‘Arteriosclerotic + heart + disease’)+OR + (‘Atherosclerosis%2 C + Coronary’)+OR + (‘Atherosclerotic + heart + disease’)+OR + (‘CAD+-+Coronary + artery + disease’)+OR + (‘CORONARY + ARTERIOSCLEROSIS’)+OR + (‘CORONARY + SCLEROSIS’)+OR + (‘Coronary + Atherosclerosis’)+OR + (‘Coronary + arteriosclerosis’)+OR + (‘Coronary + artery + arteriosclerosis’)+OR + (‘Coronary + artery + disease’)+OR + (‘Coronary + artery + sclerosis’)+OR + (‘DISEASE + CORONARY + ARTERY’)+OR + (‘DISORDER + CORONARY + ARTERY’)+OR + (‘Disease + of + the + coronary + arteries’)+OR + (‘Disease%2 C + Coronary + Artery’)+OR + (‘Disorder + of + coronary + artery’)+OR + (‘HEART%3 A + CORONARY + ARTERY’)+OR + (‘Ischaemic + heart + disease’)+OR + (‘Ischemic + heart + disease’)).

### Data classification

The data in the In-Cardiome knowledgebase haves been categorized into three classes, therapy/drug, risk factor and function in order to simplify and ease the accession of data. We used D3 scripting for visualization of data from these three classes.

*Therapy based classification.* Based on the cardiac medications classified by AHA (http://www.heart.org/HEARTORG/Conditions/HeartAttack/TreatmentofaHeartAttack/Cardiac-Medications_UCM_303937_Article.jsp#.WJxhh9J95dg) we classified the therapy/drug classes along with the drug targets and networking genes. The drugs mentioned in each therapy class were taken and their targets were identified by manual search and curating from DrugBank database (11). Based on the interacting proteins identified using STRING database (19) with already collected genes were put into specific therapy/drug class.

**Figure 2.**
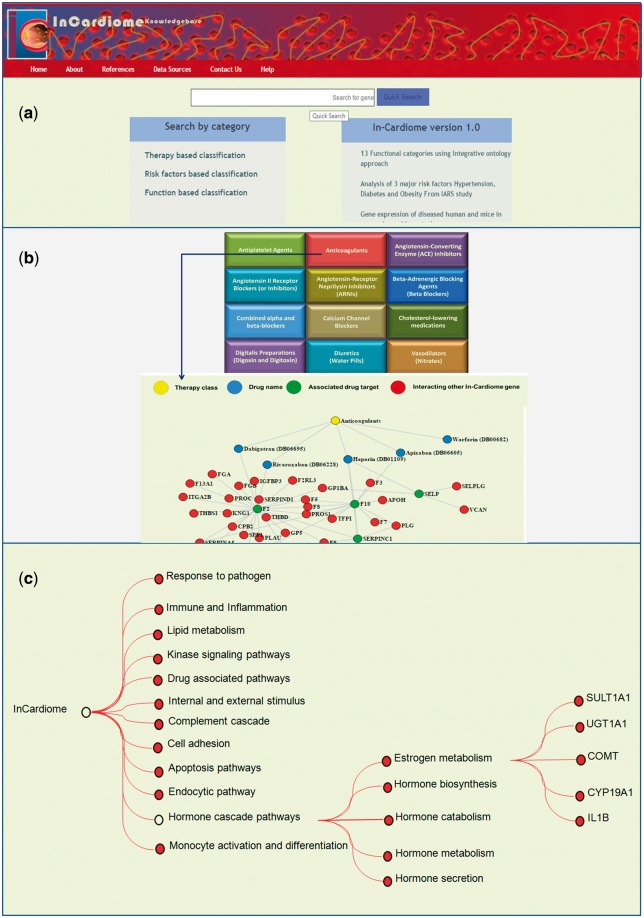
**(a)** Homepage of In-Cardiome knowledgebase. **(b)** Therapy/drug based categorization of information where upon clicking on specific therapy module, the user will be able to see the all the approved drugs, their targets and interacting proteins of those target genes. Each category has been shown with different color where yellow color circle shows drug class, blue color circle shows drug, red color circle indicates drug target and green nodes represents interacting In-Cardiome gene which may play important role in disease and therapy. **(c)** Function based classification of genes. Based on the 75 parental ontologies of 4905 GOs of 995 genes, 12 clusters were identified. Upon clicking on the any one function the user will be able to see POs and respective genes.

*Function based categorization.* We have earlier shown the identification of ‘function communicating ontologies—FCOs’ (6) which link molecular functions to genes and disease, the same approach was implemented to classify 995 genes. For all the genes, a total 4905 unique GOs were identified which grouped in to 75 parent ontologies (POs) as described by GO Consortium (geneontology.org) which were further narrowed to 12 specific functional categories or FCOs as clustered by CateGOrizer (21) (Figure 2c, Table 1). In order to show the network between specific functional categories and genes, the GO terms were matched and network was drawn using D3 script.

**Table 1.** Summery functional classification for In-Cardiome genes/proteins

Functional ontology	PO	Number of GOs	Number of genes
Response to pathogen	5	46	121
Immune and Inflammation	21	413	316
Lipid metabolism	–	166	194
Kinase signaling pathways	10	66	204
Drug associated pathways	3	6	19
Internal and external stimulus	5	423	525
Complement cascade	3	8	9
Cell adhesion	–	125	194
Apoptosis pathways	6	174	273
Endocytic pathway	–	35	103
Hormone cascade pathways	5	67	93
Monocyte activation and differentiation	3	4	6

*Risk factors-based classification.* Understanding the role of conventional risk factors like diabetes, hypertension and obesity, we classified our set of genes into 8 categories resulting from different combinations of these risk factors. The categories were none or no risk factors, hypertension, diabetes, obesity, hypertension + diabetes, hypertension + obesity, diabetes + obesity and finally hypertension + diabetes + obesity. The 995 genes of In-Cardiome were matched on to the gene sets obtained from T-HOD database (30) in order to classify into seven risk factors associated categories mentioned earlier.

*Selection of patient data from Indian Atherosclerosis Research Study* **.** Around 11 164 participants were enrolled in Indian Atherosclerosis Research Study—IARS (31) between February 2004 to June 2014, which was started primarily to understand CAD prevalence in Asian Indians and develop new biomarkers for prevention and diagnosis of the disease. IARS participants were primarily recruited from Narayana Health (formerly Narayana Hrudayalaya Hospitals), Bangalore and the Asian Heart Institute and Research Center, Mumbai. We selected 10217 subjects as rest of the participant details are still to be entered into computational database. There were 6357 unaffected subjects and 3860 CAD affected with mean age of 42.16 and 53.42, years respectively. Enrollment was done based on stringent inclusion and exclusion criteria. For patients, the age at CAD onset was ≤ 60 years in men and ≤ 65 years in women. CAD was diagnosed by coronary angiography (> 70% stenosis in major vessels or >50% stenosis in two or more smaller coronary vessels with electrocardiogram (ECG) and coronary artery bypass grafting or percutaneous coronary intervention. Clinically unaffected subjects had normal ECG readings. All subjects were free from concomitant infection or major illness at the time of enrollment with informed voluntary signed consent obtained. The entire study protocol and projects were approved by Thrombosis Research Institute Institutional Human Ethics Committee abiding by guidelines of Indian Council of Medical Research and following the principles of Declaration of Helsinki 1975. Logistic regression for odds ratio (OR) and C-statistics were performed to estimate the risk assessment of all seven risk factors combination categories (second to eighth category) with reference first. All the analysis was performed using SPSS (version 17.0).

### Data storage, construction of web interface

All the information of In-Cardiome is stored in MYSQL database by connecting different datasets using unique In-Cardiome ID as primary key and gene name as foreign key for other tables. The main table contained the gene, protein, alternative names, functions, post-translational modifications, gene expression and links to other data sources. Data regarding pathways, drug targets, GOs, phenotypes, SNP were stored in individual tables and connected using In-Cardiome ID. Furthermore, to give an easy and simple web interface, we used PHP 5.4 program. XAMPP platform was used to manage all the web services for the database. The web interface was successfully tested on Windows (XP, 7, 8, 8.1 and 10) and MAC OS SIERRA operating systems using Mozilla Firefox (version 38.0.1), Internet Explorer (version 10.0.25) and Google Chrome (version 58.0.3029.110).

## Results

### Data organization and integration

As we recognize the importance of integrating different data sources and enabling intuitive research, we selected the databases which are essential for the same. Sixteen different data sources were combined in one place with clear interlinking among them which contained 995 unique genes obtained by text mining tools (Figure 1, Supplementary Table S3). Therefore, we organized the data in two different formats for users, first we categorized all the data into three categories namely therapy/drug, function and risk factor based which can be seen on the home page (Figure 2a). This enables the user to select a specific aspect of interest for them for instance browsing the therapy/drug-based category (Figure 2b). Upon clicking on the specific therapeutic intervention, a network of drugs, their targets and interacting proteins of these targets will be displayed. We selected 12 different therapy classes as listed by AHA and universally accepted (Anticoagulants, Antiplatelet Agents, Angiotensin-Converting Enzyme (ACE) Inhibitors, Angiotensin II Receptor Blockers (or Inhibitors), Angiotensin-Receptor Neprilysin Inhibitors (ARNIs), Beta Blockers (also known as Beta-Adrenergic blocking agents), Combined alpha and beta-blockers, Calcium Channel Blockers (also known as Calcium Antagonists or Calcium Blockers), Cholesterol-lowering medications, Digitalis Preparations (also known as Digoxin and Digitoxin), Diuretics (also known as water pills) and Vasodilators (also known as Nitrates, Nitroglycerin tablets) (Table 2). The drug data also include the clinical trials information and their outcomes. Briefly, a total 62 approved Drugs and 139 drug targets were collected manually using DrugBank database and further classified into individual therapy class (Supplementary Table S4).

**Table 2.** Summery of drug class, drugs, targets and direct interacting genes

Drug class	Number of drugs	Number of targets	Number of direct interacting genes
Anticoagulants	5	4	31
ACE inhibitors	9	6	37
Angiotensin II receptor blockers (or Inhibitors)	5	3	37
ARNIs	1	1	1
Antiplatelet agents	4	5	4
Beta-adrenergic blocking agents	8	6	13
Calcium channel blockers	7	30	42
Cholesterol-lowering medications	5	9	69
Combined alpha and beta-blockers	2	14	57
Digoxin	1	1	8
Diuretics	8	44	60
Vasodilators	5	11	11

In the second method of browsing functional categorization (Figure 2c) was performed based on the GOs which are the connections between disease genes called Functional Communicating Ontologies. This resulted in 12 functional groups namely response to pathogens, immune and inflammation, lipid metabolism, kinase signaling, drug associated pathways, internal and external stimulus, complement cascade, cell adhesion, apoptosis pathways, endocytic pathways, hormone cascade pathways and finally monocyte activation and differentiation. In each functional group, enriched molecular functions and genes associated with CAD can be seen.

**Figure 3.**
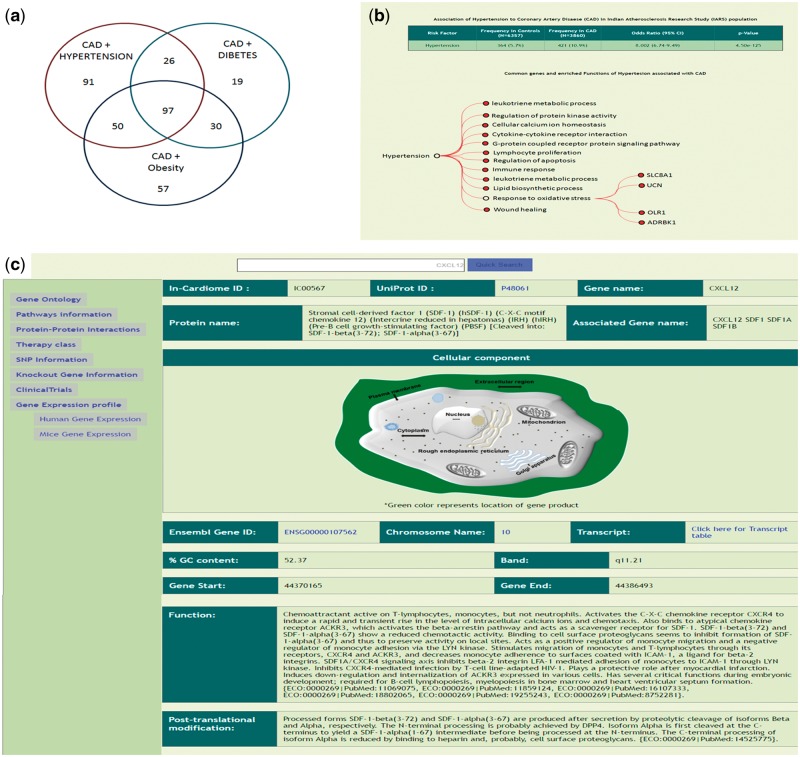
**(a)** Genes which were identified to be associated with each of the 7 combinations of risk factors as identified using T-HOD database matching of the 995 genes. **(b)** Description of Hypertensive patients and risk association of Hypertension with CAD, below enriched ontologies and respective genes are shown. **(c)** Gene specific information and also links on left side.

In the third mode of data viewing, we classified all the data into eight different classes based on the three risk factors and their combinations along with the statistical analysis for IARS population. As seen in the Figure 3a, after matching the 995 genes to T-HOD database, we identified 91 genes common between CAD and hypertension, 19 in CAD plus diabetes, 57 in CAD plus obesity, 26 in CAD + hypertension + diabetes, 30 CAD + diabetes + obesity, 50 between CAD + obesity + hypertension and finally 97 common genes between CAD + hypertension + diabetes + obesity (total of 370 genes and rest are only for CAD). The user can click on the specific FCOs associated with each of the above-mentioned categories and genes listed under that FCO (Figure 3b). The gene specific information can be seen (Figure 3c).

The unique aspect of In-Cardiome is that it provides population data on these risk factors and association to CAD in Indian population using IARS dataset. The individual risk factors, hypertension (OR: 8.002; 95% CI: 6.74-9.49; *P*-value < 0.001), diabetes (OR: 10.625; 95% CI: 8.54-13.21; *P*-value < 0.001) and obesity (OR: 2.458; 95% CI: 2.15-2.81; *P*-value < 0.001) have shown higher odds ratios with reference to those subjects who have none of these three risk factors (Table 3). While the best risk association was found with the combination of all three risk factors (category 8) (OR: 29.848; 95% CI: 24.64–36.15; *P*-value < 0.001), second highest odds ratio was found with ‘Hypertension + Diabetes’ category (OR: 15.16; 95% CI: 12.25–18.77; *P*-value < 0.001). The C-statistic analysis was performed to assess the discriminative ability this model, this model of risk factors combination showed the best performance with an AUC of 0.788 (95% CI: 0.779–797; *P*-value < 0.001). All the models built were tested for best fit using the Hosmer–Lemeshow goodness of fit test.

**Table 3.** Risk association of Individual and combined risk factors

Category	Models	Controls (*n* = 6357)	CAD affected (*n* = 3860)	Odds Ratio (95% CI)	*P*-value
1	None of the risk factors				
2	Only Hypertension	364 (5.72%)	421 (10.9%)	8.002 (6.74-9.49)	<0.001
3	Only Diabetes	168 (2.6%)	258 (6.7%)	10.62 (8.54-13.21)	<0.001
4	Only Obesity (W/H ratio > 0.93)	1776 (27.9%)	631 (16.3%)	2.458 (2.15-2.81)	<0.001
5	Hypertension + Diabetes	156 (2.5%)	342 (8.9%)	15.17 (12.25-18.77)	<0.001
6	Diabetes + Obesity	243 (3.8%)	421 (10.9%)	11.98 (9.95-14.43)	<0.001
7	Hypertension + Obesity	334 (5.3%)	578 (15.0%)	11.97 (10.13-14.14)	<0.001
8	Hypertension + Diabetes + Obesity	175 (2.8%)	755 (19.6%)	29.85 (24.64-36.15)	<0.001

### Data search

**Figure 4.**
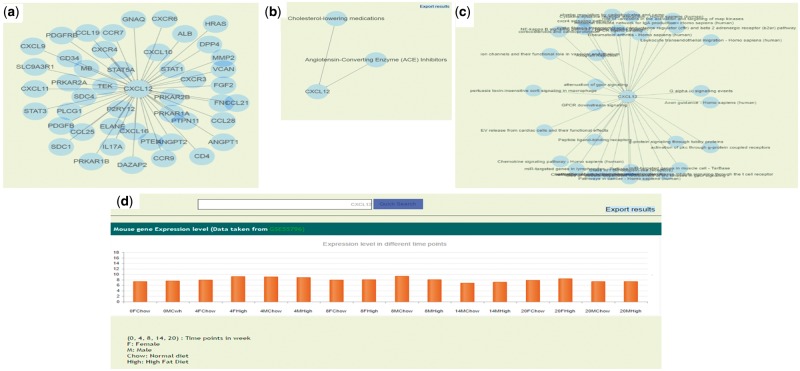
**(a)** Protein–protein interaction network of CXCL12 gene. **(b)** Gene and associated therapies suggesting potential markers for therapies. **(c)** Gene and associated pathways; **(d)** Gene expression profile of CXCL12 in ApoE^−/−^/LDLR^−/−^ double knockout mice in normal (chow diet) and high fat diet resulting in atherosclerosis in animal.

Apart from browsing the data in three unique ways, the user can search using text based search terms. For example, the user can enter specific term or gene ID or name and get the information as shown in the Figure 3c and further information can be seen by clicking on the left side links provided. The genetic information (SNP), protein-protein interactions (Figure 4a, Supplementary Table S5), GO/pathway network (Figure 4b) and therapies/drugs to gene network (Figure 4c).

The gene expression data was taken from two different datasets (GSE55796 and GSE42148). The GSE55796 dataset corresponds to animal model (ApoE^−^^/^^−^/LDLR^−^^/^^−^) experiments for normal and high fat diet conditions (Figure 4d). This study describes the gene expression profile of disease development (time points: 4, 8, 14 and 20 weeks) in mice models. Significant pathways and genes were identified with respect to the disease development (17). Similarly the gene expression profile for human control subjects (GSE42148) along with stable angina (SA) and myocardial infarction (MI) in Asian Indians was also used in the database. In this data set 190 annotated genes were differentially expressed with >2-fold, *P*-value ≤ 0.05, of which 142 were up regulated and 48 were down regulated in cases than in the controls (16). We identified 14 In-Cardiome genes to be overlapping with the up regulated genes from GSE42148 data set. Presently, we are in the process of screening more genes and the data from other ethnicity gene expression data sets that will be integrated in future versions of In-Cardiome.

## Discussion

With the current paradigm shift in the cardiovascular research towards translational research, it is mandatory that data from different sources are integrated in a logical and meaningful manner. This integration can lead to better risk prediction and therapeutic outcomes. In-Cardiome knowledgebase is primarily aimed at providing support to translational research in CAD risk prediction, biomarkers, therapeutic interventions and improved clinical outcomes. Earlier attempts like DW4TR (32) and NeuPAT (33) were useful in several ways by integrating the patient and molecular data for improved clinical applications for other diseases. DW4rt is integrated platform, providing clinical and molecular information for breast cancer disease. It uses clinical ontologies to connect patient centric information and molecular data. It provides demographic information, epidemiological information, disease pathophysiology information and gene expression information. Another unified platform NeuPAT is also an integrative clinical platform for neuroblastoma. It includes information from different sources such as clinical data, genetic and proteomic profile of patients and histopathological data combined with hospital reports. However in the field of CAD, CADgene database is one of the well-known databases. CADgene database includes very essential information of CAD associated genes such as chromosome no., gene type, protein–protein interactions and links to other resources. The data are categorized in 12 different disease associated functional categories. Similarly In-Cardiome database provides systematic integration of additional information such as clinical, molecular and epidemiological information which can give better knowledge for scientists, clinicians and pharmaceuticals. In In-Cardiome by interlinking the genomic, proteomic, interactome, pathways, expressions, drugs/therapies, clinical and animal model data a complete translational research platform has been created. Therefore, this knowledgebase can help from hypothesis generation to complete validation for clinical purposes. For example, gamma-glutamyltransferase (GGT5) search term gives all the associated information including gene expression data in animal models and human subjects. In ApoE^−^^/^^−^/LDLR^−^^/^^−^ double knockout mice, it can be observed that GGT5 expression levels were higher in fourth week high fat diet male mice in comparison to chow diet fed mice. Similarly, GGT5 was differentially expressed in patients with MI and SA than in control subjects.

Further, using this information, we have hypothesized that GGT5 may play an important role in early stage of the disease and potentially be useful for risk prediction. In our earlier research, we have validated that GGT5 is an important marker for premature cad using IARS subjects (34). Similarly the GO, pathway network and protein-protein interaction data from the search suggested that GGT5 might be an important marker linking several important functional communicator ontologies, clinical ontology of CAD and risk factors as we have shown earlier (6). Therefore, we believe that the findings and potential utilities of the database can improve the translational research.

Another example can be that the drug–target interacting proteins network can potentially be used for identification of potential leads for drug repurposing by identifying the other indication drugs which are targeting the interacting proteins of the present drug targets in the network. This is possible since these are direct interactions and can potentially have similar or desired result as those of the present targets are having. One another use could be the use of targets or interacting genes/proteins as biomarkers for clinical trials decision making. The current In-Cardiome database offers number of potential advantages as it has clinically and scientifically relevant data integrated in a logical way for ease in translational research.

### Future directions

In-Cardiome being one of its kind in supporting translational research we hope to improve this further by regular updating and adding more drug information, clinical trials analytic, meta data analysis of biomarkers and several other important aspects for research and support the dissemination of knowledge to all the healthcare community from scientists, clinicians and pharmaceutical companies.

## Supplementary data

Supplementary data are available at *Database* Online.

## Supplementary Material

Supplementary Table

Supplementary Table

Supplementary Table

Supplementary Table

Supplementary Table
